# Transoceanic origin of microendemic and flightless New Caledonian weevils

**DOI:** 10.1098/rsos.160546

**Published:** 2017-06-07

**Authors:** Emmanuel F. A. Toussaint, Rene Tänzler, Michael Balke, Alexander Riedel

**Affiliations:** 1 Florida Museum of Natural History, University of Florida, Gainesville, FL, USA; 2 SNSB-Zoological State Collection (ZSM), Münchhausenstrasse 21, 81247 Munich, Germany; 3 GeoBioCenter, Ludwig-Maximilians-University, Munich, Germany; 4 Museum of Natural History Karlsruhe (SMNK), Erbprinzenstrasse 13, 76133 Karlsruhe, Germany

**Keywords:** BioGeoBEARS, flightless beetle biogeography, Curculionidae, long-distance dispersal, Melanesia, *Trigonopterus*

## Abstract

The origin of the astonishing New Caledonian biota continues to fuel a heated debate among advocates of a Gondwanan relict scenario and defenders of late oceanic dispersal. Here, we study the origin of New Caledonian *Trigonopterus* flightless weevils using a multimarker molecular phylogeny. We infer two independent clades of species found in the archipelago. Our dating estimates suggest a Late Miocene origin of both clades long after the re-emergence of New Caledonia about 37 Ma. The estimation of ancestral ranges supports an ancestral origin of the genus in a combined region encompassing Australia and New Guinea with subsequent colonizations of New Caledonia out of New Guinea in the mid-Miocene. The two New Caledonian lineages have had very different evolutionary trajectories. Colonizers belonging to a clade of foliage dwellers greatly diversified, whereas species inhabiting leaf-litter have been less successful.

## 1. Background

**Figure 1.**
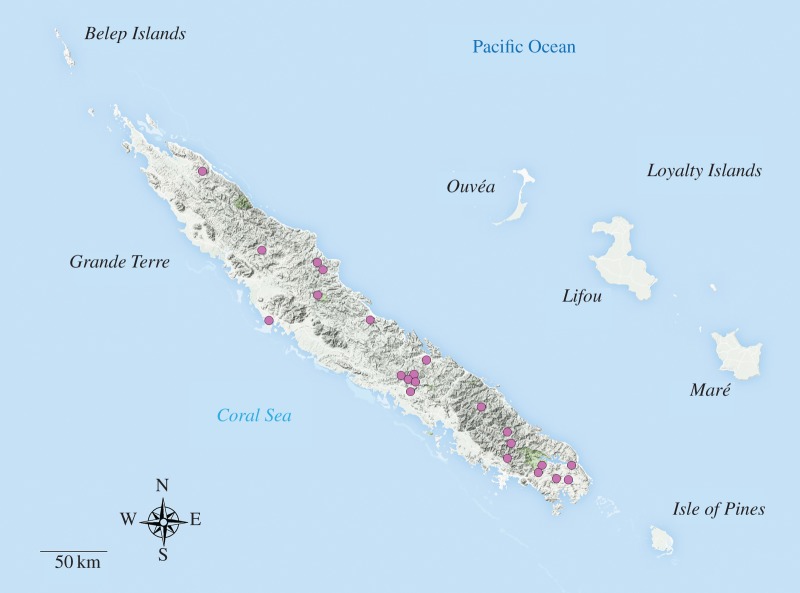
Map of New Caledonia and sampling localities. Geographical map of the New Caledonian archipelago with a relief layer indicating terrane elevation. Pink dots indicate sampling localities where specimens of *Trigonopterus* used in this study were collected (see electronic supplementary material, appendix S1 for more details).

New Caledonia is an archipelago about 1200 km east of Australia in the South Pacific (figure 1). Its main island, Grande Terre, is one of the largest Pacific islands. New Caledonia is a hotspot of biodiversity characterized by remarkable diversification and endemism, and home to a number of enigmatic or supposedly relictual lineages: *Amborella* (sister-group to the angiosperms [1]) or the Kagu of the monotypic bird family Rhynochetidae, sister-group to the South American sunbittern [2]. The origin of this biota has been intensively studied over the past century but not without significant contention [3–5]. Some authors portray New Caledonia as an ancient continental island carrying relictual Gondwanan flora and fauna to its present location and, therefore, identify diversification after vicariance as the main driver of local diversity [6–9]. The wider use of phylogenetic methods initiated a paradigm [10,11] substantiating the idea that New Caledonia is better described as an old Darwinian island and its biotic origin very well explained by transoceanic dispersal [4,12–16].

The main reason for this debate lies in the divergent interpretation of geological evidence regarding the complete submergence of New Caledonia between the Palaeocene and the Eocene [4,5]. Grande Terre is a Gondwanan fragment at the northern edge of a continental block known as Zealandia or Tasmantis, connected via the Norfolk Ridge with New Zealand in its centre, that started displacing from Gondwana *ca* 80 Ma [17]. Drifting northeastwards in the past *ca* 65 Myr, this fragment reached its present position *ca* 50 Ma. Between 65 and 45 Ma, there is clear geological evidence that Grande Terre was deeply submerged, as indicated by pelagic limestone and chert deposits [18,19]. Substantial terranes only became subaerial during an Oligocene lithosphere extension phase following the collision with the Loyalty Island arc [18–20], therefore contradicting the hypothesis of Gondwanan refuge in New Caledonia. As a result, post-Eocene dispersal commencing at *ca* 37 Ma has been invoked as the most likely scenario to explain the origin and diversification of New Caledonian clades [4]. Most of the recent studies using molecular dating to study the origin of New Caledonian clades rejected the vicariance/museum hypothesis and an associated ancient Gondwanan origin of biota (e.g. [13,21–27], but see also [28]).

However, alternative hypotheses have been proposed to explain the occurrence of relict clades in New Caledonia. Incomplete submergence of mountainous parts of Grande Terre [8] or regional dispersal from nearby islands [29,30] have been invoked to explain the ancient age of New Caledonian plants [31]. Very rare empirical examples such as the Troglosironidae harvestmen suggest a vicariant Gondwanan scenario for New Caledonian taxa [32]. In a recent study focusing on conifer macroevolution, Condamine *et al*. [33] hypothesized that now-sunk islands served as refugia for conifers when New Caledonia was submerged. This work is one of the very few examples rejecting both Cretaceous vicariance and Late Eocene dispersal as exclusive hypotheses (see also [34]). New Caledonia's biotic origins are most probably best explained by different mechanisms combined, and there remains a need for comprehensively sampled studies to investigate the origin and subsequent diversification of New Caledonian clades. Those with limited dispersal capacities might be particularly suitable, as they should be the best candidates for carrying an imprint of vicariance events.

*Trigonopterus* weevils are flightless and have a remarkable tendency towards local endemism [35]. Yet, the accumulated distribution is vast, ranging east–west from Sumatra to Samoa and south–north from New Caledonia to the Philippines. Their presence on oceanic islands (e.g. on Samoa) suggests some potential for infrequent long-distance dispersal despite their generally limited dispersal abilities. Only 90 species were described by 2012 [36,37] but hundreds of additional ones have been discovered and described since [38–40], so their current number amounts to 341 described species. *Trigonopterus* weevils are truly hyperdiverse in New Guinea, which might feature well above 1000 species [35,41]. Only very few of these appear to span ranges measuring greater than 40 km [35], and even in comparatively well-collected Australia, the majority of species are recorded only from highly restricted areas [40]. New Caledonia has 32 described species, but the same number of species or more remain undescribed [42] (A.R. 2017, unpublished data). No specimens were available from Fiji, Samoa or the Solomon Islands, from where the genus has been recorded, or Vanuatu, where it is likely to be found.

Here, we use a dated multimarker molecular phylogeny of the entire genus and a comparative biogeographical framework to test the following competing hypotheses:

1. Museum hypothesis, implying an old age >80 Ma for both the crown of the New Caledonian clade(s) and the stem node(s), with a Gondwanan origin.
2. Ancient island-hopping hypothesis, implying an old age for the stem node of the New Caledonian clade(s) but not necessarily for its crown.
3. Recent long-distance dispersal hypothesis, implying a young age <37 Ma for both the New Caledonian clade(s) and the parent node(s), with probably a Melanesian origin.

## 2. Material and methods

### 2.1. Taxon sampling and molecular biology

Besides four outgroup representatives of other Cryptorhynchinae species from Australia and New Guinea, we sampled 143 *Trigonopterus* species from their western and central geographical range, representing all important species groups and lifestyles (electronic supplementary material, appendix S1). New Caledonian *Trigonopterus* were represented by 52 species, only 18 of which could be assigned to described species. Five of the species included were each represented by two lineages possibly representing cryptic species. These 57 specimens were selected from 520 specimens collected by M. Wanat in the years 2004, 2008 and 2010, resulting in a total of 177 specimens for which a fragment of COI was sequenced. Selection of specimens and delineation of species followed procedures as outlined earlier [35,41]. A few specimens that showed no distinct morphological diagnostic characters but had a pairwise uncorrected p-distance of more than 10% were interpreted as cryptic species and included as separate species in the analysis.

Genomic DNA was extracted non-destructively using the DNeasy and NucleoSpin 96 Tissue kits (Qiagen, Hilden; Macherey-Nagel, Düren, Germany). Primers and PCR conditions are listed in electronic supplementary material, appendix S2. We sequenced nine gene fragments with an alignment of 4637 base pairs (bp) consisting of fragments from cytochrome oxidase subunit I (CO1) (2 non-overlapping fragments), mitochondrial 16S rRNA, Arginine kinase (AK), carbamoyl synthetase (CAD), Elongation factor 1α (EF1 α), Enolase (EN), histone H4 (H4) and 18S rRNA (18S).

### 2.2. Phylogenetic inference

We used IQ-TREE 1.5 [43] to infer the phylogenetic relationships among *Trigonopterus* weevils in a maximum-likelihood (ML) framework. The concatenated dataset was partitioned according to the results of a PartitionFinder v. 1.1.1 [44] analysis. The partitioning schemes and corresponding models of substitutions were searched using the *greedy* algorithm and the *raxml* set of models in PartitionFinder. The likelihoods of the different models of substitution were compared using the Akaike information criterion corrected (AICc). We performed 1000 ultrafast bootstrap replicates [45] and 1000 SH-aLRT single branch test replicates to investigate nodal support across the topology.

### 2.3. Lifestyle mapping

New Caledonian *Trigonopterus* species are found in two distinct ecological niches: (i) edaphic, i.e. dwelling in the forest litter layer and (ii) sitting on the foliage of the lower forest strata but without causing visible feeding scars [41]; these two lifestyles are very stable in species: individuals are very rarely found in the ‘wrong niche’, e.g. by specimens dropped from foliage during sifting and thus found in litter samples. Lifestyles were assigned to one of two categories (i.e. edaphic versus foliage dwellers) based on original collecting information and morphological characters (electronic supplementary material, appendix S1). They were mapped on the phylogeny using a parsimony reconstruction approach as implemented in Mesquite v. 3.2 (build 801) [46].

### 2.4. Divergence time estimates

To infer the divergence time estimates of the group, we first tested the hypothesis of the molecular clock in MEGA6 [47] by comparing the ML value of the IQ-TREE topology with and without the molecular clock constraints under the Tamura–Nei model. The null hypothesis of an equal evolutionary rate throughout the tree was rejected at a 5% significance level (*p* < 0.001). Therefore, we used a Bayesian relaxed clock approach as implemented in BEAST v. 1.8.2 [48].

‘Cryptorhynchinae *sensu latu*’ are defined by a rostral furrow which was attained by several unrelated groups, while the monophyletic Cryptorhynchinae *sensu strictu* currently cannot be diagnosed based on single morphological characters [49]. To date, there is no fossil that could be related to *Trigonopterus* and even the assignment of published records to Cryptorhynchinae s.s. is doubtful. Thus, we used two types of calibration strategies to obtain absolute divergence time estimates for the origin and further diversification of New Caledonian *Trigonopterus* weevils.

In our first strategy of calibration (CS1), we relied upon a biogeographical calibration point to enforce maximum ages in the phylogeny. In the absence of alternative reliable external calibration points (e.g. fossils), biogeographical calibrations can be very useful and provide meaningful insights [50]. Here, we used the complete submergence of Java *ca* 10 Ma [51,52] to enforce a maximum age constraint on the stem of Javanese species and/or clades in the topology. The maximum constraints were enforced in BEAUti 1.8.2 [48] using normal distributions with a mean of 10 and an s.d. of 1. The distributions were truncated to fit the interval (5–15 Ma). The use of a normal prior distribution allows considering the possibility that Javanese lineages are slightly older than the age of the re-emergence of Java *ca* 10 Ma. The concatenated dataset was partitioned by gene and each fragment was assigned a different uncorrelated lognormal relaxed clock model except for the mitochondrial fragments that were assigned a unique clock model.

In our second strategy of calibration (CS2), we used published rates of nucleotide substitution to constrain the ucld.mean (arithmetic mean of branch rates) of gene fragments sequenced in this study. A recent study used multiple fossil constraints to calculate estimates of substitution rates in different gene fragments sequenced for *Carabus* ground beetles [53]. This is one of the most recent, fossil-based studies providing ucld.mean estimates and credibility intervals for different gene fragments in flightless beetles. This study recovered a ucld.mean of 0.0134 substitutions per site per million year and per lineage (credibility interval CI = 0.0108–0.0162 subst/site/Myr/lin). A different study [54] conducted on flightless darkling beetles (Coleoptera, Tenebrionidae) recovered comparable rates for the mitochondrial clock using a well-documented biogeographic calibration point (ucld.mean = 0.0119 subst/site/Myr/lin; CI = 0.0108–0.0140 subst/site/Myr/lin). Considering the assumption that mitochondrial genes are following a same clock model, we constrained the mitochondrial ucld.mean in our dataset (CO1 and 16S combined), with a uniform prior distribution encompassing the CI (0.0108–0.0162 subst/site/Myr/lin), calculated for mitochondrial genes of *Carabus* beetles [53]. We assumed a different clock for each nuclear gene fragment, and the ucld.mean estimates of these were left unconstrained.

In both analyses, the tree model was set to a Yule model, and we used the IQ-TREE topology as a fixed input by editing the .xml file. The runs consisted of 30 million generations sampled every 1000 generations for CS1, and of 50 million generations sampled every 5000 generations for CS2. Convergence of the runs was assessed by checking the likelihood trace of each parameter and its effective sample size in Tracer v. 1.6 (http://BEAST.bio.ed.ac.uk/Tracer). A value of ESS >200 was acknowledged as a good indicator of convergence. The maximum credibility consensus tree, median ages and their 95% highest posterior density (HPD) were generated afterwards under TreeAnnotator v. 1.8.2 [48].

### 2.5. Biogeographical analyses

**Figure 2.**
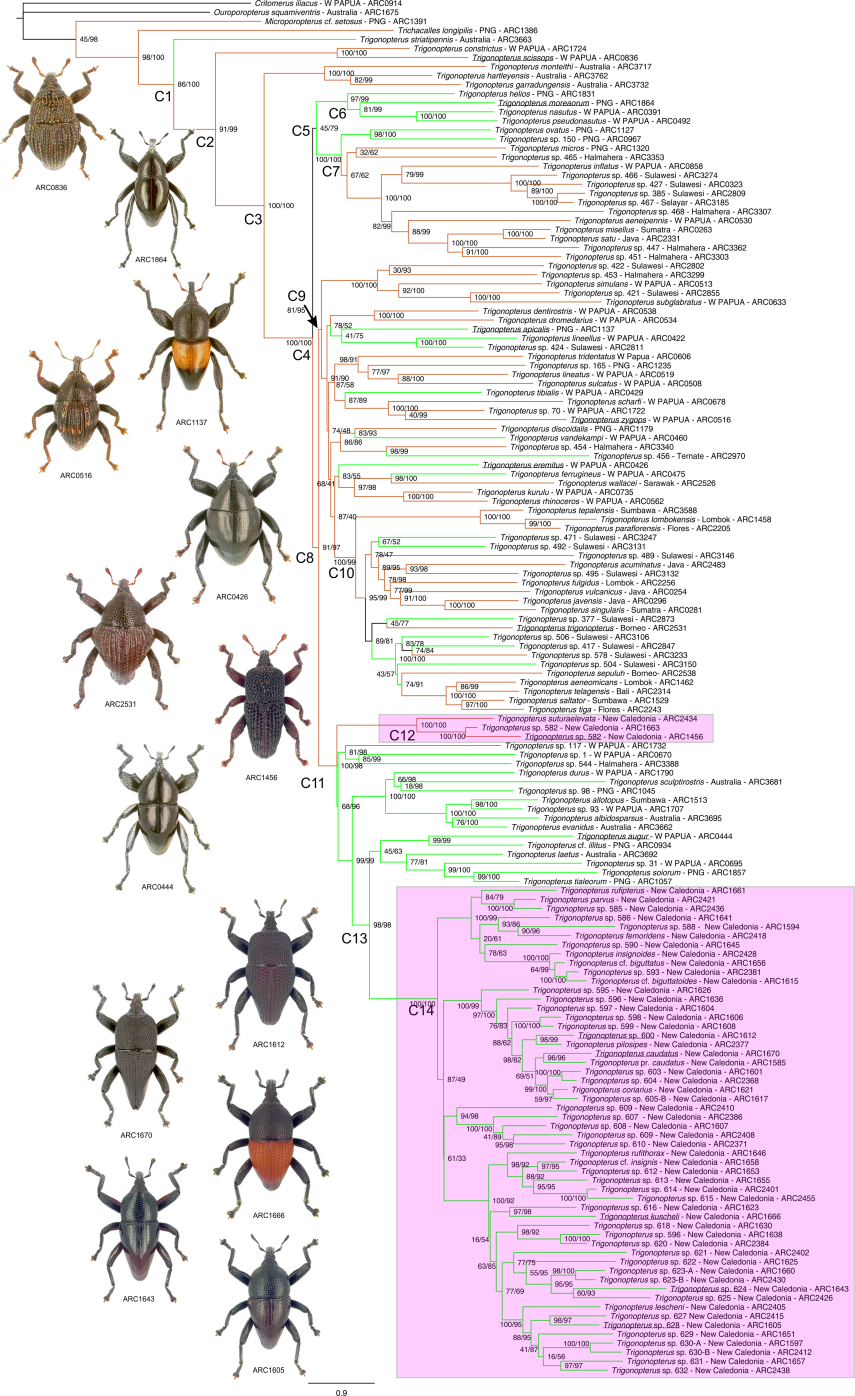
Phylogenetic tree of New Caledonian *Trigonopterus*. Maximum-likelihood phylogeny inferred using IQ-TREE. The main clades are labelled. Support values of nodes are given with SH-aLRT (left) and ultrafast bootstrap (right). Brown lineages indicate an edaphic lifestyle, and green lineages indicate foliage dwellers; black lineages are with an ambiguous lifestyle . Pink boxes mark New Caledonian taxa. Labels of species illustrated to the left are underlined.

We used BioGeoBEARS [55] as implemented in R to estimate the ancestral ranges in the *Trigonopterus* phylogeny. This software implements three different biogeographic models (DEC, DIVALIKE and BAYAREALIKE) in a comparative framework. It also allows taking into account founder-event jump dispersal when the parameter + *j* is included in the model. The analyses were carried out based on the BEAST maximum clade credibility (MCC) tree (electronic supplementary material, S1) derived from the CS1 analysis (see Results and discussion), and with out-groups removed. We used the following regions in the analyses: A, Australia; B, Borneo; C, New Caledonia; G, New Guinea; J, Java; L, Lesser Sunda islands; M, Moluccas; S, Sumatra and W, Sulawesi (figure 2). As the Indo-Australian archipelago has a very dynamic geological history, we designed two time slices to take into account this palaeogeographic evolution [51,52]. In the first time slice (30–15 Ma), the Australian and Oriental regions are still separated by a large water corridor, whereas in a second time slice (15–0 Ma), the complex assemblage of the Wallacea is already starting, thereby connecting the two regions. The dispersal rate scalers for each time slice were calculated based on geographical distance between areas as the archipelago was forming through time. Before 15 Ma, we restricted dispersal between each side of the ocean (Oriental region/Australian region) to long-distance dispersal (rate scaler of 0.01). After 15 Ma, when the Asian and Australian plates collided and the orogeny of the archipelago initiated [15,51], the rate scalers were based on the distance of the regions from one another, with values proportionally decreasing with the increasing distance of focal areas (see electronic supplementary material, appendix S3 for scaler matrices in the two time slices). Considering the complexity of the landscape and the relative proximity of terranes across the archipelago [52], we allowed all possible area combinations in the adjacency matrices of the two time slices. The maximum number of joint areas was limited to three in all analyses.

## 3. Results and discussion

**Figure 3.**
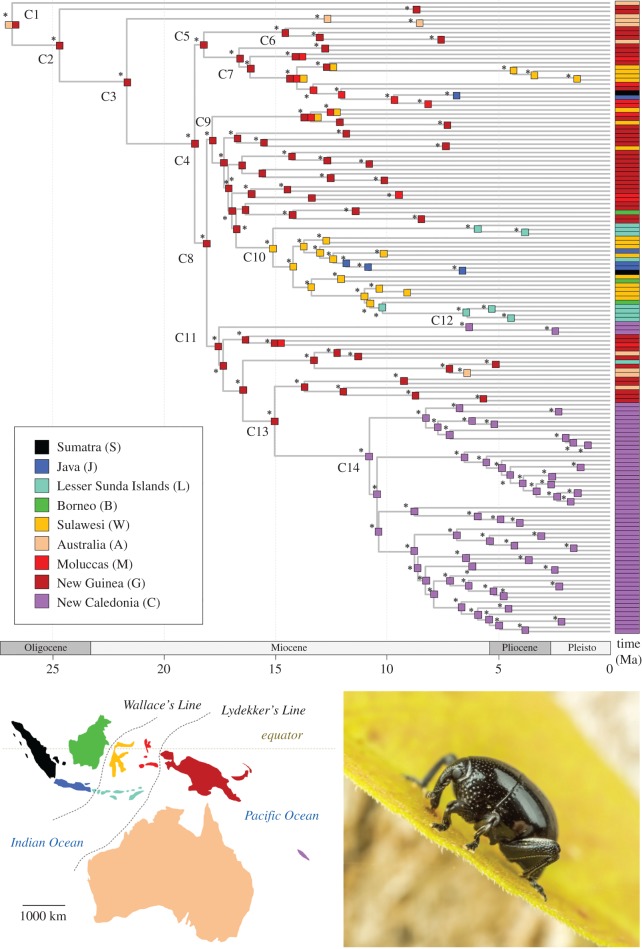
Palaeobiogeography of New Caledonian *Trigonopterus*. BEAST chronogram presenting median age estimates for the *Trigonopterus* radiation (see electronic supplementary material, appendix S4 for the full chronogram with CI bars). The main clades are labelled as in figure 2 and their age estimates are detailed in table 1. For each species used in this study, the distribution as defined in the BioGeoBEARS analyses is given at the corresponding tip. The colours are coded following the map inserted at the bottom left corner of the figure and as indicated in the caption. The ancestral range estimation recovered in the preferred BioGeoBEARS analyses (DIVALIKE + *j*) is presented for every node of the phylogeny. Asterisks indicate that the estimated best ancestral range had more than 50% relative probability compared with other possible ancestral ranges (see electronic supplementary material, appendix S6 for more details). The photograph inserted at the bottom right corner highlights a specimen of *Trigonopterus* in natura (Queensland, Australia, Photo D. Yarrow).

*Trigonopterus* was retrieved as monophyletic and the topology of our reconstruction is well resolved (figure 2), with mostly high ultrafast bootstrap values for the main nodes of the backbone. The New Caledonian species are unambiguously placed in two separate clades with strong support. The BEAST analyses conducted with the two different calibration strategies gave congruent divergence time estimates (table 1). The results of the BEAST dating analysis using the Java submergence calibration (CS1) are summarized in figure 3, with details for major clade nodes in table 1 (see electronic supplementary material, appendix S4 for more details). The best model in the BioGeoBEARS biogeographical analyses is the DIVALIKE + *j* although the DEC + *j* model has an almost identical likelihood (table 2). Both models reveal a very similar pattern with slight differences in regions of the tree that are, however, not the focus of this study (electronic supplementary material, appendix S5). In the DIVALIKE + *j* estimation, the ancestral range estimation was unambiguous, as preferred ancestral ranges for each node of the topology had a significantly better likelihood (greater than 50% of relative probability) than the next possible ancestral range (figure 3; electronic supplementary material, appendix S6). Our estimates place the origin of the genus at *ca* 27 Ma in a joint Australia + New Guinea ancestral region (clade C1). Most of the lineages then occupied New Guinea from where species dispersed westwards, starting in the Mid-Miocene (clade C10). This early occurrence in New Guinea deviates from recent geological evidence suggesting a more recent emergence of New Guinea, although some amount of land and possibly even altitude might have been available at the time in a proto-Papuan archipelagic setting [15,52,56–58]. Even small geographical areas, as long as they have some higher elevations, might feature a rather diverse *Trigonopterus* fauna as was shown for the Cyclops Mountains of Papua with 54 species found mainly along a single transect [35,41].

**Table 1.** Median ages of major *Trigonopterus* clades as inferred in BEAST. CS1, calibration strategy 1 with Java biogeographical calibration points; CS2, calibration strategy 2 with the *Carabus* mitochondrial substitution rate interval; all ages are in Myr.

	CS1 age (95 CI)	CS2 median age (95 CI)
clade C1	26.60 (23.27–30.09)	25.77 (20.60–32.70)
clade C2	24.41 (21.23–27.29)	23.55 (18.87–28.84)
clade C3	21.21 (18.91–24.28)	20.85 (16.73–26.24)
clade C4	18.53 (16.48–20.47)	18.20 (14.76–22.61)
clade C5	18.08 (16.02–20.25)	17.18 (13.88–21.38)
clade C6	15.33 (13.10–17.50)	13.78 (10.29–18.22)
clade C7	16.18 (14.32–18.33)	15.32 (12.24–19.09)
clade C8	18.04 (16.12–19.96)	17.52 (14.31–21.79)
clade C9	17.76 (15.71–19.65)	17.07 (13.82–21.10)
clade C10	15.06 (13.29–17.07)	14.00 (11.34–17.52)
clade C11	17.58 (15.55–19.41)	16.55 (13.46–20.51)
clade C12	6.38 (5.10–9.25)	6.07 (3.98–8.54)
clade C13	15.09 (13.37–16.99)	13.90 (11.30–17.33)
clade C14	10.88 (9.68–12.22)	10.88 (8.88–13.50)

**Table 2.** Results of the BioGeoBEARS comparative analyses.

model	LnL	parameters	*d*	*e*	*j*	percentage Akaike weight
DEC	303.98	2	0.100	0.100	0.000	0.000
DEC + *j*	186.609	3	0.007	0.000	0.156	49.900
DIVALIKE	207.581	2	0.043	0.001	0.000	0.000
DIVALIKE + *j*	186.608	3	0.010	0.000	0.140	49.950
BAYAREALIKE	238.909	2	0.038	0.035	0.000	0.000
BAYAREALIKE + *j*	192.441	3	0.001	0.001	0.188	0.150

The lineages found west of Wallace's Line originated from dispersal out of New Guinea in the mid-Miocene as estimated in clade C7 and clade C10, the latter having used Sulawesi as a stepping stone. This is in line with palaeogeological reconstructions of the region [51,52] suggesting a junction of the Asian and Australian plates at this time, therefore facilitating short-distance dispersal. The dating also matches the transgression of Wallace's Line in Sundaic *Trigonopterus* clades [16,58] and other weevil clades [59].

The two New Caledonian clades C12 and C14 are found to be of at least Mid-to-Late Miocene origin and presumably originated from New Guinea via long-distance dispersal. The actual lineage diversification in New Caledonia began *ca* 6 Ma and *ca* 10 Ma in C12 and C14, respectively.

Lifestyles of *Trigonopterus* species (i.e. edaphic versus foliage) are phylogenetically relatively stable (figure 2). Changes are over-represented in our phylogeny because we tried to maximize diversity in our species selection by choosing representatives of each group for each lifestyle. Both lifestyles are represented in New Caledonia, and they are phylogenetically congruent with the two lineages: species of clade 12 are edaphic, while those of clade 14 are all found on foliage. This might indicate some undersampling of the edaphic fauna, but extensive litter sifting in New Caledonia by Geoff Monteith only revealed two additional species (in the Queensland Museum, Brisbane). Thus, the observed pattern seems to reflect the actual relative species diversity in the two clades, one species poor and the other one with marked diversification after colonization. The same pattern was also found in New Caledonian *Exocelina* diving beetles [60,61]; in that case a species-poor older clade and a diverse younger one, with the older species in marginal habitats, thus possibly indicating replacement. The diversification of *Trigonopterus* in New Caledonia was relatively late in the context of other lineages of the genus, well after New Guinea and Sulawesi (figure 3), and more comparable to the Lesser Sunda Islands.

Both New Caledonian clades have comparably long branches, which might be explained by (i) extinction of ancestral species on New Caledonia or the now-submerged land nearby or (ii) lack of sampling in New Caledonia or the Solomon Islands, Vanuatu, Fiji and Samoa. We can, however, partially reject the ancient island-hopping hypothesis. While the long branches potentially allow for an island-hopping scenario, this would not predate the proposed Late Eocene emergence of Grand Terre. We also reject an ancient origin of New Caledonian *Trigonopterus* and thus the museum hypothesis, as their main diversification was fast and during the past 10 Myr, whereas the museum hypothesis would require a crown and a diversification of New Caledonian *Trigonopterus* over at least 80 Ma [31]. Even if our biogeographic dating analysis is not optimal (but see [50]), the differences between our estimates and the requirements for a Gondwanan origin are so distinct that we can here reject such a scenario. Instead, we suggest a recent long-distance dispersal with a Melanesian origin for the New Caledonian *Trigonopterus*. Dispersal of these weevils across deep water straits (e.g. Wallace's Line) has in fact been robustly documented previously [16,58]. The actual dispersal mechanism for these flightless beetles remains to be tested empirically, but it might involve larvae floating safely contained in or between branches or logs [16]. The small body size of *Trigonopterus* weevils (1.5–6.0 mm, but usually 2–4 mm) could also favour other dispersal mechanisms. However, a lifestyle of inhabiting the lower vegetation or the leaf-litter, plus their compact body and their heavy chitinization make their dispersal by strong winds highly improbable. External attachment to birds appears rather impossible, though birds may act as vector if the weevils are ingested inside fruits or seeds. All this is somewhat speculative and the currently unknown larval development of *Trigonopterus* weevils would be important information to let us choose among hypotheses. Additional sampling from nearby archipelagoes (Solomons, Vanuatu) located between New Guinea and New Caledonia would complete our understanding of the biogeographical history of this endemic fauna. There may be some faunal exchange between New Caledonia and these islands. In fact, New Caledonia may have played a role as a stepping stone to the Pacific islands further east. However, the fauna of New Guinea and Australia clearly emerged as the cradle of *Trigonopterus* evolution: basal diversification extended for more than 10 Myr in this area before other islands to the west and east were colonized. All Australian clades representing the foliage-dwelling lifestyle are contained in the present analysis; they are somewhat scattered among the equally foliage-dwelling Papuan species of clade C11 and containing the foliage-dwelling clade C14 of New Caledonia. The absence of the edaphic *T. australis* group of three species from the tip of Cape York [40] may require a later re-interpretation of the origin of New Caledonian clade C12 to which it may be related. Past extinction events of the Australian fauna may be missing in this scenario and are hard to consider with the absence of fossils. A situation as presented by the subgenus *Brassospora* of *Nothofagus* [62] cannot be ruled out and a colonization of New Caledonia from Northern Australia could in fact be a possibility, although not supported by our present analysis. In any case, long-range transoceanic dispersal set the stage for a later radiation of *Trigonopterus* in New Caledonia. The wide distribution across the Indomalayan Archipelago and across Wallace's and Lydekker's Lines suggests that transport over open ocean was not uncommon during the evolution of *Trigonopterus* [16].

In summary, our study suggests that transoceanic dispersal of flightless animals and their subsequent fast diversification is one factor contributing to New Caledonia's exceptional diversity.

## Supplementary Material

Supplementary Information

## Data accessibility

Voucher specimens are currently kept in the museum collections of SMNK (State Museum of Natural History Karlsruhe, Germany) and MNHW (Museum of Natural History, Wrocław University, Poland); some may later be transferred to MNHN (Museum national d'Histoire naturelle, Paris, France) if they become holotypes. The alignment of DNA sequence data used for the analysis was deposited at Dryad: http://dx.doi.org/10.5061/dryad.vq31g [63]. Data on genetic material contained in this paper have been submitted to EMBL and accession numbers are given in electronic supplementary material, appendix S1. These sequence data are published for non-commercial use only. Utilization for purposes other than non-commercial scientific research may infringe the conditions under which the genetic resources were originally accessed, and should not be undertaken without contacting the corresponding author of the paper and/or seeking permission from the original provider of the genetic material.
