# Supplementary material for: Transoceanic origin of microendemic and flightless New Caledonian weevils
Source: R Soc Open Sci. 2017 Jun 7;4(6):160546. doi: 10.1098/rsos.160546 (PMC5493895; doi:10.1098/rsos.160546)

Supplementary Information

Oceanic Origin of Microendemic and Flightless New Caledonian Weevils

Appendix 1. Taxon sampling

Appendix 2. Table of primers and PCR protocols

Appendix 3. Dispersal matrices used in the BioGeoBEARS analyses

Appendix 4. BEAST chronogram with median ages and 95% height posterior distributions

Appendix 5. Results of the different BioGeoBEARS analyses.The regions used in the analyses are as follow: A, Australia (red); B, Borneo (pink); C, New Caledonia (light blue); G, New Guinea (dark blue); J, Java (yellowish green); L, Lesser Sunda islands (green); M, Moluccas (blueish green); S, Sumatra (orange); and W, Sulawesi (purple).

Appendix 6. Detailed result of the DIVALIKE+j analysis conducted in BioGeoBEARS

Specimen-Code	Genus	Species	Authority	Area	Locality	Life-Style	PatJerry	ArgK	H4	EF1a	18S	16S	CAD 3	EN
ARC0254	Trigonopterus	vulcanicus	Riedel	Java	W-Java, Telaga Warna, between Puncak and Cipanas	edaphic	HG939629	HG939762	HG940108	HG939982	HG940281	HG940207	HG939910	HG940041
ARC0263	Trigonopterus	misellus	Riedel	Sumatra	E-Sumatra, Lampung, Pedada Bay, Bawang, Gn. Tanggang	edaphic	LN885002			LN884669			LN880351	LN884746
ARC0281	Trigonopterus	singularis	Marshall	Sumatra	E-Sumatra, Lampung, Kalianda, Gn. Rajabasa	edaphic	LN885005	LN884584		LN884671		LN884325	LN880355	LN884749
ARC0296	Trigonopterus	javensis	Riedel	Java	C-Java, Petungkriyono, N slopes of Dieng plateau, Mountain N of Tinalum	edaphic	LN885007	LN884585	LN884850	LN884674			LN880357	LN884751
ARC0323	Trigonopterus	sp. 427		Sulawesi	N-Sulawesi, Bitung, Gn. Dua Saudara	edaphic			LN884853	LN884677	LN884422	LN884328		
ARC0391	Trigonopterus	nasutus	(Pascoe)	New Guinea	W-Papua, Manokwari Prov., Manokwari, Gn. Meja	foliage	LT603147	LT603221	LT603302	LT603351		LT603465	LT603550	LT603627
ARC0422	Trigonopterus	lineellus	Riedel	New Guinea	W-Papua, Jayapura Reg., Sentani, Cyclops Mts.	foliage			HG940112	HG939984	HG940286	HG940211	HG939915	HG940046
ARC0426	Trigonopterus	eremitus	Riedel	New Guinea	W-Papua, Jayapura Reg., Sentani, Cyclops Mts.	foliage	HG939634	HG939765	HG940113	HG939985	HG940287	HG940212	HG939916	HG940047
ARC0429	Trigonopterus	tibialis	Riedel	New Guinea	W-Papua, Jayapura Reg., Sentani, Cyclops Mts.	foliage	HG939635	HG939766	HG940114		HG940288	HG940213	HG939917	HG940048
ARC0444	Trigonopterus	augur	Riedel	New Guinea	W-Papua, Jayapura Reg., Sentani, Cyclops Mts.	foliage		LT603222			LT603395	LT603466		LT603628
ARC0460	Trigonopterus	vandekampi	Riedel	New Guinea	W-Papua, Jayapura Reg., Sentani, Cyclops Mts.	foliage	HG939636	HG939767		HG939986	HG940289	HG940214	HG939918	HG940049
ARC0475	Trigonopterus	ferrugineus	Riedel	New Guinea	W-Papua, Jayapura Reg., Sentani, Cyclops Mts.	foliage	HG939637	HG939768		HG939987	HG940290	HG940215	HG939919	HG940050
ARC0492	Trigonopterus	pseudonasutus	Riedel	New Guinea	W-Papua, Jayapura Reg., Sentani, Cyclops Mts.	foliage	HG939638	HG939769	HG940115	HG939988	HG940291	HG940216	HG939920	HG940051
ARC0508	Trigonopterus	sulcatus	Riedel	New Guinea	W-Papua, Jayapura Reg., Sentani, Cyclops Mts.	edaphic	HG939639	HG939770	HG940116	HG939989	HG940292		HG939921	HG940052
ARC0513	Trigonopterus	simulans	Riedel	New Guinea	W-Papua, Jayapura Reg., Sentani, Cyclops Mts.	edaphic	LT603148	LT603223		LT603352		LT603467	LT603551	
ARC0516	Trigonopterus	zygops	Riedel	New Guinea	W-Papua, Jayapura Reg., Sentani, Cyclops Mts.	edaphic	HG939640	HG939771	HG940117	HG939990	HG940293	HG940217	HG939922	HG940053
ARC0519	Trigonopterus	lineatus	Riedel	New Guinea	W-Papua, Jayapura Reg., Sentani, Cyclops Mts.	edaphic		HG939772	HG940118	HG939991	HG940294	HG940218	HG939923	HG940054
ARC0530	Trigonopterus	aeneipennis	Riedel	New Guinea	W-Papua, Jayapura Reg., Sentani, Cyclops Mts.	edaphic	HG939641			HG939992	HG940295	HG940219	HG939924	HG940055
ARC0534	Trigonopterus	dromedarius	Riedel	New Guinea	W-Papua, Jayapura Reg., Sentani, Cyclops Mts.	edaphic		HG939773		HG939993	HG940296	HG940220	HG939925	HG940056
ARC0538	Trigonopterus	dentirostris	Riedel	New Guinea	W-Papua, Jayapura Reg., Sentani, Cyclops Mts.	edaphic		HG939774	HG940119	HG939994	HG940297	HG940221	HG939926	HG940057
ARC0562	Trigonopterus	rhinoceros	Riedel	New Guinea	W-Papua, Jayapura Reg., Sentani, Cyclops Mts.	edaphic	HG939642	HG939775	HG940120	HG939995	HG940298		HG939927	HG940058
ARC0606	Trigonopterus	tridentatus	Riedel	New Guinea	W-Papua, Manokwari, Gn. Meja	edaphic	HG939647		HG940125	HG939999	HG940304			HG940063
ARC0633	Trigonopterus	subglabratus	Riedel	New Guinea	W-Papua, Jayapura Reg., Sentani, Cyclops Mts.	edaphic	LT603149		LT603303	LT603353		LT603468		
ARC0670	Trigonopterus	cf. illex	(Faust)	New Guinea	W-Papua, Jayapura Reg., Sentani, Cyclops Mts.	foliage	HG939648	HE613866	HG940126	HG940000	HG940305	HG940226	HG939933	HG940064
ARC0678	Trigonopterus	scharfi	Riedel	New Guinea	W-Papua, Jayapura Reg., Sentani, Cyclops Mts.	edaphic	HG939649	HG939780	HG940127	HG940001	HG940306	HG940227	HG939934	
ARC0695	Trigonopterus	sp. 31		New Guinea	W-Papua, Jayapura Reg., Sentani, Cyclops Mts.	foliage	HG939650	HG939781	HG940128	HG940002	HG940307	HG940228		
ARC0735	Trigonopterus	kurulu	Riedel	New Guinea	W-Papua, Jayawijaya Reg., Jiwika, Kurulu	edaphic	HG939653	HG939783		HG940005	HG940310	HG940230	HG939936	HG940065
ARC0836	Trigonopterus	scissops	Riedel	New Guinea	W-Papua, Manokwari Reg., Arfak Mts., Mokwam, Siyoubrig	edaphic	HG939654	HG939784	HG940130	HG940006	HG940311	HG940231	HG939937	HG940066
ARC0858	Trigonopterus	inflatus	Riedel	New Guinea	W-Papua, Biak Reg., Biak Island, Nernu	edaphic	HG939655	HG939785	HG940131		HG940312	HG940232	HG939938	HG940067
ARC0914	Critomerus	iliacus	(Pascoe)	New Guinea	W-Papua, Jayapura Reg., Sentani, Cyclops Mts.	ambiguous	HG939656	HG939786	HG940132	HG940007	HG940313		HG939939	LN884755
ARC0934	Trigonopterus	cf. illitus	(Faust)	New Guinea	PNG, Central Prov., Varirata N.P.	foliage		LT603224	LT603304			LT603469	LT603552	
ARC0967	Trigonopterus	sp.150		New Guinea	PNG, Central Prov., Moroka area, Kailaki, Mt. Berogoro	foliage	HG939657	HG939787	HG940133	HG940008	HG940314	HG940233	HG939940	HG940068
ARC1045	Trigonopterus	sp. 98		New Guinea	PNG, Central Prov., Moroka area, Kailaki, Beremutana ridge	foliage	LT603150					LT603470	LT603553	
ARC1057	Trigonopterus	tialeorum	Riedel	New Guinea	PNG, Eastern Highlands Prov., Goroka, Mt. Gahavisuka	foliage	LT603151	LT603225				LT603471	LT603554	
ARC1127	Trigonopterus	ovatus	Riedel	New Guinea	PNG, Simbu Prov., Karimui Dist., Haia, Supa	foliage	LT603152	LT603226	LT603305		LT603396	LT603472	LT603555	LT603629
ARC1137	Trigonopterus	apicalis	Riedel	New Guinea	PNG, Simbu Prov., Karimui Dist., Haia, Supa	foliage	LT603153	LT603227				LT603473	LT603556	LT603630
ARC1179	Trigonopterus	discoidalis	Riedel	New Guinea	PNG, Morobe Prov., Huon peninsula, Mindik	edaphic	HG939658	HG939788	HG940134		LN884423	HG940234	HG939941	HG940069
ARC1235	Trigonopterus	sp. 165		New Guinea	PNG, Morobe Prov., Huon peninsula, mountain SW Pindiu	foliage	HG939659		HG940135		HG940315	HG940235		
ARC1320	Trigonopterus	micros	Riedel	New Guinea	PNG, Morobe Prov., Huon peninsula, Sattelberg	edaphic	HG939660	HG939789	HG940136	HG940009	HG940316	HG940236		
ARC1386	Acalles (Trichacalles)	longipilis	Voss	New Guinea	PNG, Morobe Prov., Huon peninsula, Sattelberg	edaphic	LN889603	LN888814		LN889060	LN888462	LN888284	LN888954	
ARC1391	Microporopterus	cf. setosus	Voss	New Guinea	PNG, Morobe Prov., Huon peninsula, Sattelberg	edaphic	HG939661	HG939790	HG940137	HG940010	HG940317	HG940237	HG939942	
ARC1456	Trigonopterus	sp. 582, pr. suturaelevata	Rheinheimer	New Caledonia	New Caledonia, Cap Ndoua, site 2, berleseate	edaphic	LT603154		LT603306			LT603474		
ARC1458	Trigonopterus	lombokensis	Riedel	Lesser Sunda Islands	Lombok Isl., Tetebatu, Rinjani-trail from Orong Gerisak	edaphic	HG939664	HG939792	HG940138		HG940320	HG940240	HG939944	HG940071
ARC1462	Trigonopterus	aeneomicans	Riedel	Lesser Sunda Islands	Lombok Isl., Tetebatu, Rinjani-trail from Orong Gerisak	edaphic	HG939666	HG939794	HG940140	HG940013	HG940322	HG940242	HG939946	HG940073
ARC1513	Trigonopterus	allotopus	Riedel	Lesser Sunda Islands	W-Sumbawa, Batu Dulang, Gn. Batu Pasak	foliage		HG939801	HG940147	HG940020	HG940329	HG940249	HG939953	
ARC1529	Trigonopterus	saltator	Riedel	Lesser Sunda Islands	W-Sumbawa, Batu Dulang, Gn. Batu Pasak	edaphic	HG939674	HG939803	HG940148	HG940022	HG940331	HG940251	HG939955	HG940080
ARC1585	Trigonopterus	caudatus	Heller	New Caledonia	New Caledonia, Bois du Sud	foliage	LT603155	LT603228	LT603307		LT603397	LT603475	LT603557	
ARC1594	Trigonopterus	sp. 588		New Caledonia	New Caledonia, Pic du Pin (base)	foliage	LT603156	LT603229	LT603308	LT603354	LT603398	LT603476	LT603558	LT603631
ARC1597	Trigonopterus	coriarius	Heller	New Caledonia	New Caledonia, Koghi Mts; roadside, ex *Hibbertia lucens*	foliage	LT603157	LT603230	LT603309	LT603355	LT603399	LT603477	LT603559	
ARC1601	Trigonopterus	sp. 603, pr. hassleri	Rheinheimer	New Caledonia	New Caledonia, Koghi Mts; forest entrance	foliage	LT603158	LT603231	LT603310		LT603400	LT603478	LT603560	LT603632
ARC1604	Trigonopterus	sp. 597, pr. hassleri	Rheinheimer	New Caledonia	New Caledonia, Dzumac Mts., road from jct to old mine	foliage	LT603159	LT603232	LT603311		LT603401	LT603479		
ARC1605	Trigonopterus	sp. 628, pr. sculpturatus	Rheinheimer	New Caledonia	New Caledonia, Dzumac Mts., road from jct to old mine	foliage	LT603160	LT603233	LT603312	LT603356	LT603402	LT603480	LT603561	
ARC1606	Trigonopterus	sp. 598, pr. setipoides	Rheinheimer	New Caledonia	New Caledonia, Dzumac Mts., Mt. Quin road jct, day beating	foliage	LT603161	LT603234			LT603403	LT603481	LT603562	
ARC1607	Trigonopterus	sp. 608		New Caledonia	New Caledonia, Dzumac Mts., Mt. Quin road jct, day beating	foliage	LT603162	LT603235			LT603404	LT603482	LT603563	LT603633
ARC1608	Trigonopterus	sp. 599, pr. setipoides	Rheinheimer	New Caledonia	New Caledonia, Dzumac Mts., Mt. Quin road jct, day beating	foliage		LT603236		LT603357	LT603405	LT603483	LT603564	LT603634
ARC1612	Trigonopterus	sp. 600, pr. pilosipes	Rheinheimer	New Caledonia	New Caledonia, Dzumac road, forest, night beating roadside	foliage	LT603163	LT603237	LT603313		LT603406	LT603484	LT603565	
ARC1615	Trigonopterus	biguttoides ?	Rheinheimer	New Caledonia	New Caledonia, Pic Ningua, roadside vegetation	foliage	LT603164	LT603238		LT603358	LT603407	LT603485	LT603566	LT603635
ARC1617	Trigonopterus	sp. 605-B, pr. hassleri	Rheinheimer	New Caledonia	New Caledonia, Mt. Do, roadside, night beating	foliage	LT603165	LT603239			LT603408	LT603486	LT603567	LT603636
ARC1621	Trigonopterus	sp. 605-A, pr. hassleri	Rheinheimer	New Caledonia	New Caledonia, Humboldt, S track	foliage	LT603166	LT603240		LT603359	LT603409	LT603487	LT603568	
ARC1623	Trigonopterus	sp. 616, pr. rufipterus	Rheinheimer	New Caledonia	New Caledonia, Humboldt, S track, botanical reserve, moss forest	foliage	LT603167	LT603241	LT603314	LT603360	LT603410	LT603488	LT603569	
ARC1625	Trigonopterus	sp. 622, pr. diversicollis	Heller	New Caledonia	New Caledonia, Humboldt, S track, botanical reserve, moss forest	foliage	LT603168	LT603242		LT603361	LT603411	LT603489	LT603570	LT603637
ARC1626	Trigonopterus	sp. 595		New Caledonia	New Caledonia, Humboldt, S track, maquis, beating down to Col du Vulcain	foliage	LT603169	LT603243	LT603315		LT603412	LT603490	LT603571	LT603638
ARC1630	Trigonopterus	sp. 618, pr. sexmaculatus	Rheinheimer	New Caledonia	New Caledonia, Col d´Amieu (3.0 km to gate)	foliage	LT603170	LT603244	LT603316	LT603362	LT603413	LT603491	LT603572	LT603639
ARC1636	Trigonopterus	hassleri ?	Rheinheimer	New Caledonia	New Caledonia, Col d´Amieu (3.0 km to gate)	foliage	LT603171	LT603245	LT603317		LT603414	LT603492	LT603573	LT603640
ARC1638	Trigonopterus	sp. 619		New Caledonia	New Caledonia, Col d´Amieu (3.0 km to gate)	foliage	LT603172	LT603246			LT603415	LT603493	LT603574	
ARC1641	Trigonopterus	sp. 586		New Caledonia	New Caledonia, Mandjélia	foliage	LT603173	LT603247		LT603363	LT603416	LT603494	LT603575	LT603641
ARC1643	Trigonopterus	sp. 624, pr. insignoides	Rheinheimer	New Caledonia	New Caledonia, Mandjélia	foliage	LT603174	LT603248	LT603318		LT603417	LT603495	LT603576	
ARC1645	Trigonopterus	sp. 590, similar to caudatus	Heller	New Caledonia	New Caledonia, Mandjélia, track -> pic 1 km NW summit	foliage	LT603175	LT603249	LT603319		LT603418	LT603496	LT603577	LT603642
ARC1646	Trigonopterus	rufithorax	Rheinheimer	New Caledonia	New Caledonia, Mandjélia, track -> pic 1 km NW summit	foliage		LT603250		LT603364	LT603419	LT603497	LT603578	
ARC1651	Trigonopterus	sp. 629, pr. coriarius	Heller	New Caledonia	New Caledonia, Pic d´Amoa (Povila), road end	foliage	LT603176	LT603251			LT603420	LT603498	LT603579	
ARC1653	Trigonopterus	sp. 612		New Caledonia	New Caledonia, Aoupinié, up of gravelpit	foliage	LT603177	LT603252			LT603421	LT603499	LT603580	LT603643
ARC1655	Trigonopterus	biguttatus ?	Rheinheimer	New Caledonia	New Caledonia, Aoupinié, up of gravelpit	foliage	LT603178	LT603253	LT603320		LT603422	LT603500	LT603581	
ARC1656	Trigonopterus	quadrimaculatus	Rheinheimer	New Caledonia	New Caledonia, Aoupinié, up of gravelpit	foliage	LT603179	LT603254		LT603365	LT603423	LT603501	LT603582	LT603644
ARC1657	Trigonopterus	sp. 631, pr. coriarius	Heller	New Caledonia	New Caledonia, Aoupinié, up of gravelpit	foliage	LT603180	LT603255			LT603424	LT603502	LT603583	
ARC1658	Trigonopterus	cf. insignis	Fauvel	New Caledonia	New Caledonia, Aoupinié, up Goipin road junction	foliage		LT603256			LT603425	LT603503	LT603584	LT603645
ARC1660	Trigonopterus	sp. 623-A		New Caledonia	New Caledonia, Aoupinié, up Goipin road junction	foliage	LT603181	LT603257		LT603366	LT603426	LT603504	LT603585	LT603646

ARC1661	Trigonopterus	rufipterus	Rheinheimer	New Caledonia	New Caledonia, 8 km SSE Poro, plateau above mine, ex *Hibbertia lucens*	foliage	LT603182	LT603258		LT603367	LT603427	LT603505	LT603586	LT603647
ARC1663	Trigonopterus	sp. 582, pr. suturaelevata	Rheinheimer	New Caledonia	New Caledonia, Forêt Nord, rainforest, sifting	edaphic	LT603183	LT603259	LT603321	LT603368	LT603428	LT603506	LT603587	LT603648
ARC1666	Trigonopterus	kuscheli	Rheinheimer	New Caledonia	New Caledonia, Col de Yate, highest point, maquis	foliage	LT603184	LT603260	LT603322	LT603369	LT603429	LT603507	LT603588	LT603649
ARC1670	Trigonopterus	caudatus	Heller	New Caledonia	New Caledonia, Col des Deux Tétons	foliage	LT603185	LT603261	LT603323		LT603430	LT603508	LT603589	LT603650
ARC1675	Ouroporopterus	squamiventris	Lea	Australia	Queensland, Lamington N.P., IBISCA 700A, Malaise, RF	ambiguous	HG939677	HG939806	HG940151		HG940334	HG940254	HG939956	
ARC1707	Trigonopterus	sp. 93		New Guinea	W-Papua, Jayawijaya Reg., Jiwika, Kurulu	foliage	HG939678		HG940152	HG940025	HG940335	HG940255	HG939957	
ARC1722	Trigonopterus	sp. 70		New Guinea	W-Papua, Jayawijaya Reg., Poga	edaphic	HG939679				HG940336	HG940256	HG939958	HG940083
ARC1724	Trigonopterus	constrictus	Riedel	New Guinea	W-Papua, Jayawijaya Reg., Poga	edaphic	LT603186	LT603262	LT603324	LT603370		LT603509		
ARC1732	Trigonopterus	sp. 117		New Guinea	W-Papua, Jayawijaya Reg., Poga	edaphic			HG940153	HG940026	HG940337	HG940257	HG939959	HG940084
ARC1790	Trigonopterus	durus	Riedel	New Guinea	W-Papua, Jayawijaya Reg., Bokondini	foliage	HG939680		HG940154	LN884681	HG940338	HG940258	HG939960	HG940085
ARC1831	Trigonopterus	helios	Riedel	New Guinea	PNG, Eastern Highlands Prov., Okapa, Kimiagomo vilage, Afiyaleto	foliage	LT603187	LT603263	LT603325			LT603510		
ARC1857	Trigonopterus	soiorum	Riedel	New Guinea	PNG, Eastern Highlands Prov., Aiyura	foliage	LT603188	LT603264				LT603511		
ARC1864	Trigonopterus	moreaorum	Riedel	New Guinea	PNG, Eastern Highlands Prov., Okapa, Kimiagomo vilage, Verefare	foliage	LT603189	LT603265	LT603326	LT603371		LT603512	LT603590	LT603651
ARC2205	Trigonopterus	paraflorensis	Riedel	Lesser Sunda Islands	Flores Isl., Ruteng, Gn. Ranaka	edaphic		LN884594	LN884861	LN884685		LN884335	LN880367	LN884764
ARC2243	Trigonopterus	tiga	Riedel	Lesser Sunda Islands	Flores Isl., Labuhan Bajo, Tebedo	edaphic	HG939687	HG939813	HG940160		HG940346	HG940266	HG939968	HG940093
ARC2256	Trigonopterus	fulgidus	Riedel	Lesser Sunda Islands	Lombok Isl., Gn. Rinjani, Senaru, Rinjani-track	edaphic	HG939690	HG939815	HG940163		HG940349	HG940269	HG939971	HG940096
ARC2314	Trigonopterus	telagensis	Riedel	Lesser Sunda Islands	Bali Isl., Busungbiu – Pupuan, Telaga, Primatani reserve	edaphic	HG939691	HG939817	HG940165		HG940351	HG940271	HG939973	HG940098
ARC2331	Trigonopterus	satu	Riedel	Java	East Java, Banyuwangi Reg., Meru Betiri N.P., Sukamade	edaphic		LN884597	LN884863			LN884338	LN880370	LN884767
ARC2368	Trigonopterus	sp. 604, pr. hassleri	Rheinheimer	New Caledonia	New Caledonia, Col des Deux Tétons	foliage	LT603190	LT603266	LT603327		LT603431	LT603513	LT603591	LT603652
ARC2371	Trigonopterus	diversicollis ?	Heller	New Caledonia	New Caledonia, Col des Deux Tétons	foliage	LT603191	LT603267		LT603372	LT603432	LT603514	LT603592	LT603653
ARC2377	Trigonopterus	pilosipes	Rheinheimer	New Caledonia	New Caledonia, Pic du Pin (base)	foliage	LT603192	LT603268	LT603328	LT603373	LT603433	LT603515	LT603593	LT603654
ARC2381	Trigonopterus	sp. 593		New Caledonia	New Caledonia, Pic du Grand Kaori	foliage	LT603193	LT603269		LT603374	LT603434	LT603516	LT603594	LT603655
ARC2384	Trigonopterus	sp. 620		New Caledonia	New Caledonia, Parc des Grandes Fougères, N of Pic Vincent	foliage	LT603194	LT603270	LT603329		LT603435	LT603517	LT603595	LT603656
ARC2386	Trigonopterus	sp. 607		New Caledonia	New Caledonia, Parc des Grandes Fougères, N of Pic Vincent	foliage	LT603195	LT603271	LT603330		LT603436	LT603518	LT603596	LT603657
ARC2401	Trigonopterus	pseudoinsignis	Rheinheimer	New Caledonia	New Caledonia, Sarramea, trail to Dogny	foliage	LT603196	LT603272	LT603331	LT603375	LT603437	LT603519	LT603597	LT603658
ARC2402	Trigonopterus	sp. 621, pr. heteropunctatus	Rheinheimer	New Caledonia	New Caledonia, Sarramea, trail to Dogny	foliage	LT603197	LT603273			LT603438	LT603520	LT603598	LT603659
ARC2405	Trigonopterus	lescheni	Rheinheimer	New Caledonia	New Caledonia, Sarramea, trail to Dogny	foliage		LT603274		LT603376	LT603439	LT603521	LT603599	
ARC2408	Trigonopterus	sp. 609		New Caledonia	New Caledonia, Sarramea, trail to Dogny	foliage	LT603198	LT603275	LT603332	LT603377	LT603440	LT603522	LT603600	LT603660
ARC2410	Trigonopterus	sp. 606		New Caledonia	New Caledonia, Sarramea, trail to Dogny	foliage	LT603199	LT603276	LT603333	LT603378	LT603441	LT603523	LT603601	
ARC2412	Trigonopterus	coriarius ?	Heller	New Caledonia	New Caledonia, S Humboldt trail	foliage	LT603200	LT603277		LT603379	LT603442	LT603524	LT603602	
ARC2415	Trigonopterus	sp. 627, pr. dichrous	Montrouzier	New Caledonia	New Caledonia, Pindai Peninsula, Sentier de la Laverie, sclerophyll forest	foliage		LT603278	LT603334	LT603380	LT603443	LT603525	LT603603	LT603661
ARC2418	Trigonopterus	femoridens	Rheinheimer	New Caledonia	New Caledonia, Pic d´Amoa, Povilla, rainforest	foliage	LT603201	LT603279	LT603335		LT603444	LT603526	LT603604	LT603662
ARC2421	Trigonopterus	parvus	Rheinheimer	New Caledonia	New Caledonia, Pic d´Amoa, Povilla, rainforest	foliage	LT603202	LT603280		LT603381	LT603445	LT603527	LT603605	
ARC2426	Trigonopterus	sp. 625, pr. insignoides	Rheinheimer	New Caledonia	New Caledonia, Pic d´Amoa, Povilla, rainforest	foliage	LT603203	LT603281	LT603336		LT603446	LT603528	LT603606	
ARC2428	Trigonopterus	insignoides	Rheinheimer	New Caledonia	New Caledonia, Pic d´Amoa, Povilla, rainforest	foliage	LT603204	LT603282		LT603382	LT603447	LT603529	LT603607	LT603663
ARC2430	Trigonopterus	sp. 623-B		New Caledonia	New Caledonia, Pic d´Amoa, Povilla, rainforest	foliage	LT603205	LT603283		LT603383	LT603448	LT603530	LT603608	
ARC2434	Trigonopterus	suturaelevata	Rheinheimer	New Caledonia	New Caledonia, Sarramea, trail to Dogny, sifting litter nr. Wye riv. pass	edaphic	LT603206	LT603284		LT603384	LT603449	LT603531	LT603609	LT603664
ARC2436	Trigonopterus	sp. 585		New Caledonia	New Caledonia, Canala, 4 km S of Mia, rd end, river valley, rainforest	foliage	LT603207	LT603285		LT603385	LT603450	LT603532	LT603610	
ARC2438	Trigonopterus	sp. 632, pr. coriarius	Heller	New Caledonia	New Caledonia, Canala, 4 km S of Mia, rd end, river valley, rainforest	foliage	LT603208	LT603286	LT603337	LT603386	LT603451	LT603533	LT603611	
ARC2455	Trigonopterus	sp. 615, pr. insignoides	Rheinheimer	New Caledonia	New Caledonia, Col des Roussettes, rainforest E of river	foliage		LT603287	LT603338		LT603452	LT603534	LT603612	LT603665
ARC2483	Trigonopterus	acuminatus	Riedel	Java	E-Java, Gn. Wilis, Kediri, Besuki	edaphic	HG939694	HG939820	HG940167	HG940036	HG940354			HG940101
ARC2526	Trigonopterus	wallacei	Riedel	Borneo	Sarawak, Kuching, Mt. Santubong	edaphic		HG939822	HG940169	HG940038	HG940356	HG940275	HG939977	
ARC2531	Trigonopterus	trigonopterus	Riedel	Borneo	W-Kalimantan, Bengkayan, Suka-Bangun, Gn. Bawang	edaphic	HG939696	HG939823	HG940170		HG940357	HG940276	HG939978	HG940103
ARC2538	Trigonopterus	sepuluh	Riedel	Borneo	W-Kalimantan, Bengkayan, Suka-Bangun, Gn. Bawang	edaphic	HG939697	HG939824	HG940171		HG940358	HG940277	HG939979	HG940104
ARC2802	Trigonopterus	sp. 422		Sulawesi	N-Sulawesi, Airmadidi, Gn. Klabat	edaphic	LT603209	LT603288	LT603339			LT603535	LT603613	LT603666
ARC2809	Trigonopterus	sp. 385		Sulawesi	SE-Sulawesi, Wakatobi, Wangi Wangi Isl., Matahora	edaphic	LT603210	LT603289	LT603340	LT603387		LT603536	LT603614	LT603667
ARC2811	Trigonopterus	sp. 424		Sulawesi	S-Sulawesi, Pc. Palopo, Gn. Sampuna	foliage	LT603211	LT603290	LT603341			LT603537	LT603615	LT603668
ARC2847	Trigonopterus	sp. 417		Sulawesi	C-Sulawesi, Pendolo, Gn. Sampuraga	foliage		LN884619				LN884362	LN880392	LN884789
ARC2855	Trigonopterus	sp. 421		Sulawesi	C-Sulawesi, Pendolo, Gn. Sampuraga	foliage	LN885032	LN884621	LN884886	LN884703	LN884426	LN884364	LN880394	LN884790
ARC2873	Trigonopterus	sp. 377		Sulawesi	C-Sulawesi, Tentena, Taripa	foliage	LN885035	LN884624	LN884887	LN884706	LN884428	LN884367	LN880397	LN884793
ARC2970	Trigonopterus	sp. 456		Moluccas	Ternate Isl., Marikurubu, Gn. Gamalama	foliage	LN885040	LN884628		LN884708	LN884433	LN884372	LN880402	LN884797
ARC3106	Trigonopterus	sp. 506		Sulawesi	C-Sulawesi, Pendolo, Boe	foliage	LN885051	LN884638	LN884899		LN884444	LN884384	LN880414	LN884809
ARC3131	Trigonopterus	sp. 492		Sulawesi	C-Sulawesi, Pendolo, Gn. Sampuraga	foliage	LN885053		LN884901	LN884718	LN884446	LN884386		LN884811
ARC3132	Trigonopterus	sp. 495		Sulawesi	C-Sulawesi, Pendolo, Boe	edaphic	LN885054	LN884639	LN884902	LN884719	LN884447	LN884387	LN880416	LN884812
ARC3146	Trigonopterus	sp. 489		Sulawesi	SE-Sulawesi, Kendari, Road from Wawotobi to Lasolo	edaphic	LN885058		LN884905		LN884451	LN884391	LN880420	LN884816
ARC3150	Trigonopterus	sp. 504		Sulawesi	SE-Sulawesi, Kendari, Road from Wawotobi to Lasolo	foliage	LN885059	LN884642			LN884452	LN884392	LN880421	LN884817
ARC3185	Trigonopterus	sp. 467		Sulawesi	Selayar Isl., Pagarangan	edaphic	LT603212	LT603291	LT603342	LT603388	LT603453	LT603538	LT603616	
ARC3233	Trigonopterus	sp. 578		Sulawesi	S-Sulawesi, Mangkutana	edaphic	LN885069	LN884650	LN884913	LN884727	LN884463	LN884402	LN880432	LN884826
ARC3247	Trigonopterus	sp. 471		Sulawesi	C-Sulawesi, Ampana, Tanjung Api	edaphic	LN885072	LN884653	LN884915		LN884466		LN880435	LN884829
ARC3274	Trigonopterus	sp. 466		Sulawesi	C-Sulawesi, Luwuk, Salodi, Gn. Taluanjang	edaphic		LN884655	LN884917	LN884730	LN884468	LN884405	LN880437	
ARC3299	Trigonopterus	sp. 453		Moluccas	Halmahera Isl., Kobe, Weda	edaphic	LN885076	LN884656			LN884471	LN884408	LN880440	LN884832
ARC3303	Trigonopterus	sp. 451		Moluccas	Halmahera Isl., Kobe, Weda	edaphic	LN885078		LN884921		LN884473	LN884410	LN880442	LN884834
ARC3307	Trigonopterus	sp. 468		Moluccas	Halmahera Isl., Weda, road to Sofifi	edaphic			LN884922	LN884732	LN884474	LN884411	LN880443	
ARC3340	Trigonopterus	sp. 454		Moluccas	Halmahera Isl., Buli	edaphic	LT603213			LT603389	LT603454	LT603539	LT603617	
ARC3353	Trigonopterus	sp. 465		Moluccas	Halmahera Isl., Buli	edaphic		LT603292	LT603343		LT603455	LT603540	LT603618	LT603669
ARC3362	Trigonopterus	sp. 447		Moluccas	Halmahera Isl., Buli, road to Subaim	edaphic	LT603214		LT603344		LT603456	LT603541	LT603619	LT603670
ARC3388	Trigonopterus	sp. 544		Moluccas	Halmahera Isl., Jailolo, Payo, Gn. Jailolo	foliage		LT603293	LN884927	LN884736	LN884480	LN884417	LN880447	LN884837
ARC3588	Trigonopterus	tepalensis	Riedel	Lesser Sunda Islands	W-Sumbawa, Tepal, Pc. Nengas	edaphic	LN885083	LN884661	LN884928	LN884737		LN884418		LN884838
ARC3662	Trigonopterus	sp. 571		Australia	Queensland, Brisbane, St. Lucia	foliage	LT603215	LT603294	LT603345	LT603390	LT603457	LT603542	LT603620	LT603671
ARC3663	Trigonopterus	sp. 550		Australia	Queensland, North Stradbroke Isl., 3,5 km SW Point Lookout, Fishermans Road	foliage		LT603295	LT603346		LT603458	LT603543		LT603672
ARC3681	Trigonopterus	sculptirostris	Lea	Australia	Queensland, Mission Beach, Clump Mt. N.P., Bicton Hill	foliage	LT603216	LT603296	LT603347	LT603391	LT603459	LT603544	LT603621	
ARC3692	Trigonopterus	laetus	Lea	Australia	Queensland, Mt. Cook N.P., Cooktown	foliage	LT603217	LT603297	LT603348		LT603460	LT603545	LT603622	LT603673
ARC3695	Trigonopterus	albidosparsus	Lea	Australia	Queensland, Mt. Cook N.P., Cooktown	foliage		LT603298	LT603349	LT603392	LT603461	LT603546	LT603623	LT603674
ARC3717	Trigonopterus	monteithi	Riedel	Australia	Queensland, Kuranda N.P., Saddle Mountain Road	edaphic	LT603218	LT603299	LT603350	LT603393	LT603462	LT603547	LT603624	LT603675
ARC3732	Trigonopterus	garradungensis	Riedel	Australia	Queensland, Garradunga, Polly Ck., N Innisfail, Hasenpusch property	edaphic	LT603219	LT603300			LT603463	LT603548	LT603625	LT603676
ARC3762	Trigonopterus	hartleyensis	Riedel	Australia	Queensland, Cedar Bay N.P., road between Rossville and Bloomfield	edaphic	LT603220	LT603301		LT603394	LT603464	LT603549	LT603626	LT603677

Gene	Location	Primer	Direction	Sequence (5' -> 3')	PCR protocol	References
Cytochrome c oxidase 1; 5´end	mitochondrial	LCO1490-JJ	FOR	CHACWAAYCATAAAGATATYGG	D:94°C for 30 sec; A:47(52)°C for 40 sec; E:72°C for 60 sec	Astrin & Stüben, 2008
		HCO2198-JJ	REV	AWACTTCVGGRTGVCCAAARAATCA	5 cycles 47°C, 30 cycles 52°C	Astrin & Stüben, 2008
Cytochrome c oxidase 1; 3´ end		Jerry	FOR	CAACATTTATTTTGATTTTTTGG	D:94°C for 30 sec; A:47(52)°C for 40 sec; E:72°C for 60 sec	Simon et al., 1994
		Pat	REV	TCCAATGCACTAATCTGCCATATTA	5 cycles 47°C, 30 cycles 52°C	Simon et al., 1994
16S		1472-JJ	FOR	AGATAGAAACCRACCTGG	D:94°C for 30 sec; A:47(52)°C for 40 sec; E:72°C for 60 sec	Astrin & Stüben, 2008
		ar-JJ	REV	CRCCTGTTTATTAAAAACAT	5 cycles 47°C, 30 cycles 52°C	Astrin & Stüben, 2008
18S	nuclear	18S 5'	FOR	GACAACCTGGTTGATCCTGCCAGT	D:94°C for 30 sec; A:47(52)°C for 40 sec; E:72°C for 60 sec	Shull et al., 2001
		18S b5.0	REV	TAACCGCAACAACTTTAAT	5 cycles 47°C, 30 cycles 52°C	Shull et al., 2001
CAD3		CD821F	FOR	AGCACGAAAATHGGNAGYTCNATGAARAG	D:94°C for 30 sec; A:53°C for 30 sec; E:72°C for 60 sec	Wild & Maddison, 2008
		CD1098R2	REV	GCTATGTTGTTNGGNAGYTGDCCNCCCAT	35 cycles	Wild & Maddison, 2008
Enolase		EN37F	FOR	GACTCTCGTGGNAAYCCNACNGTNGAGGT	D:94°C for 30 sec; A:53°C for 30 sec; E:72°C for 60 sec	Wild & Maddison, 2008
		EN731R	REV	CTTGTAGAACTCNGANGCNGCNACRTCCAT	35 cycles	Wild & Maddison, 2008
Arginine kinase		AK183F	FOR	GATTCTGGAGTCGGNATYTAYGCNCCYGAYGC	D:94°C for 30 sec; A:53°C for 30 sec; E:72°C for 60 sec	Wild & Maddison, 2008
		AK939R	REV	GCCNCCYTCRGCYTCRGTGTGYTC	35 cycles	Wild & Maddison, 2008
Elongation factor 1α		efs372	FOR	CTGGTGAATTTGAAGCYGGTA	D:94°C for 30 sec; A:58-42°C for 30 sec; E:72°C for 60 sec	McKenna et al., 2005
		efa754	REV	CCACCAATTTTGTAGACATC	ΔT -2°C every 3 cycles (58-44°C); 18 cycles 42°C	Normark et al., 1999
Histone 4		H4F2s	FOR	TSCGIGAYAACATYCAGGGIATCAC	D:94°C for 30 sec; A:49°C for 30 sec; E:72°C for 60 sec	Pineau et al., 2004
		H4F2er	REV	CKYTTIAGIGCRTAIACCACRTCCAT	35 cycles	Pineau et al., 2004

Appendix 3. Dispersal matrices used in the BioGeoBEARS analyses

30-15	A	B	J	L	M	C	G	W	S
A	1	0.1	0.01	0.01	0.01	0.1	0.01	0.01	0.1
B	0.1	1	0.01	0.01	0.01	0.1	0.01	0.01	1
J	0.01	0.01	1	0.01	0.01	0.1	0.01	0.01	0.01
L	0.01	0.01	0.01	1	0.01	0.1	0.01	0.01	0.01
M	0.01	0.01	0.01	0.01	1	0.1	0.01	0.01	0.01
C	0.1	0.1	0.1	0.1	0.1	1	0.01	0.01	0.1
G	0.01	0.01	0.01	0.01	0.01	0.01	1	0.01	0.01
W	0.01	0.01	0.01	0.01	0.01	0.01	0.01	1	0.01
S	0.1	1	0.01	0.01	0.01	0.1	0.01	0.01	1

15-0	A	B	J	L	M	C	G	W	S
A	1	0.25	0.25	0.5	0.5	0.1	1	0.5	0.25
B	0.25	1	1	0.5	0.5	0.1	0.25	0.5	1
J	0.25	1	1	1	0.5	0.1	0.25	0.5	1
L	0.5	0.5	1	1	0.5	0.1	0.5	1	0.5
M	0.5	0.5	0.5	0.5	1	0.1	1	1	0.25
C	0.1	0.1	0.1	0.1	0.1	1	0.1	0.1	0.1
G	1	0.25	0.25	0.5	1	0.1	1	0.5	0.25
W	0.5	0.5	0.5	1	1	0.1	0.5	1	0.5
S	0.25	1	1	0.5	0.25	0.1	0.25	0.5	1

Appendix 4. BEAST chronogram with median ages and 95% height posterior distributions

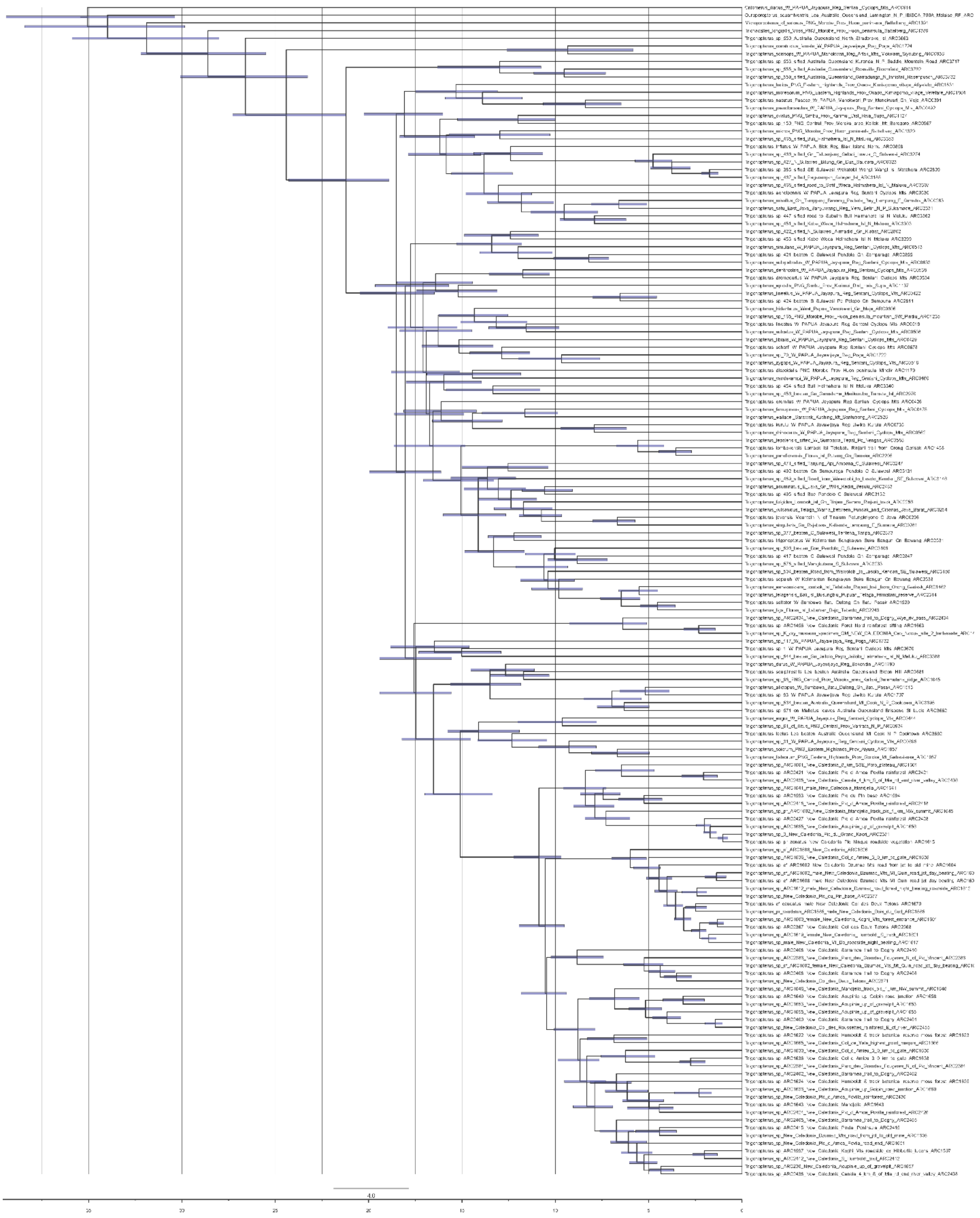

Appendix 5. Results of the different BioGeoBEARS analyses.

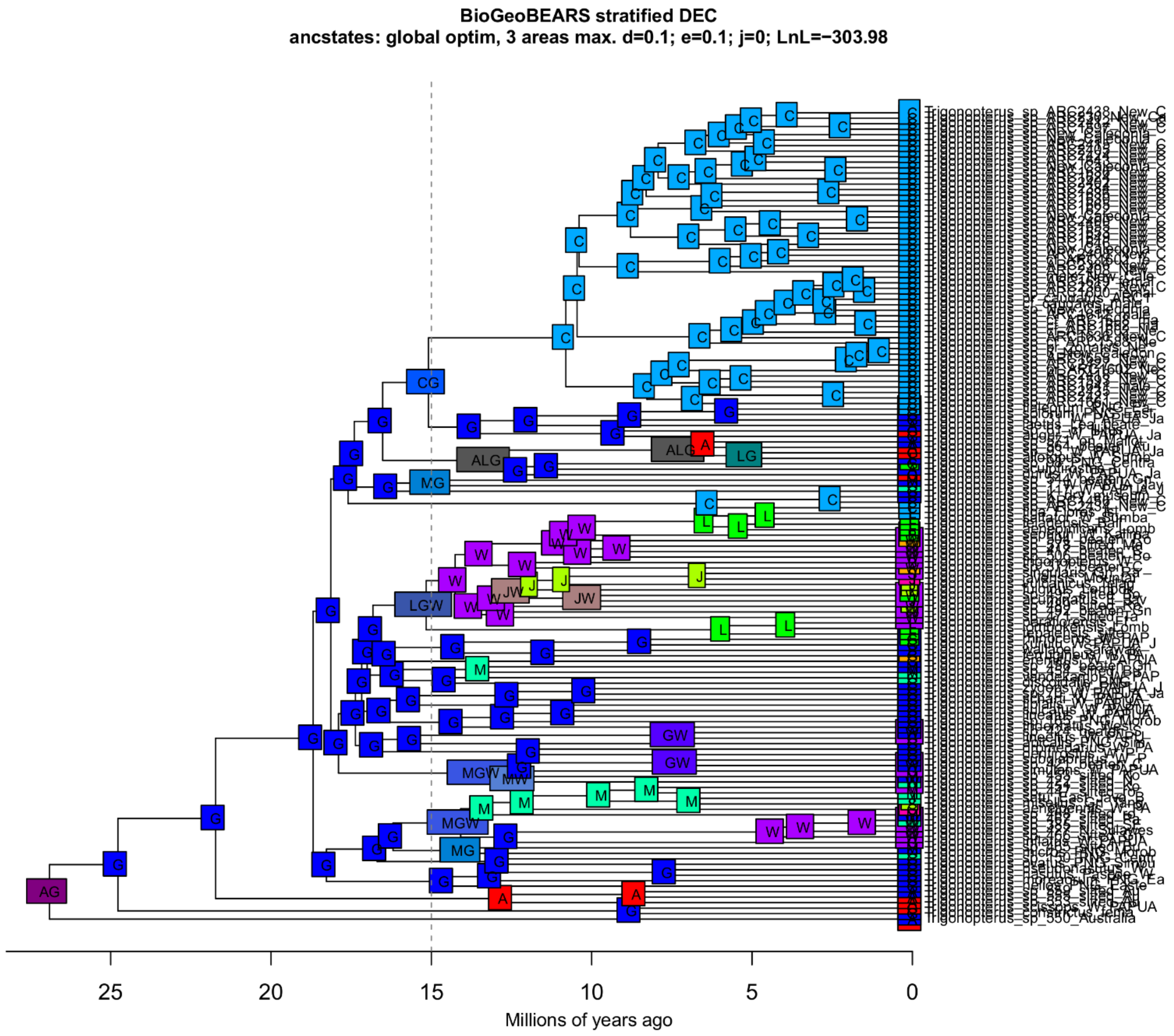

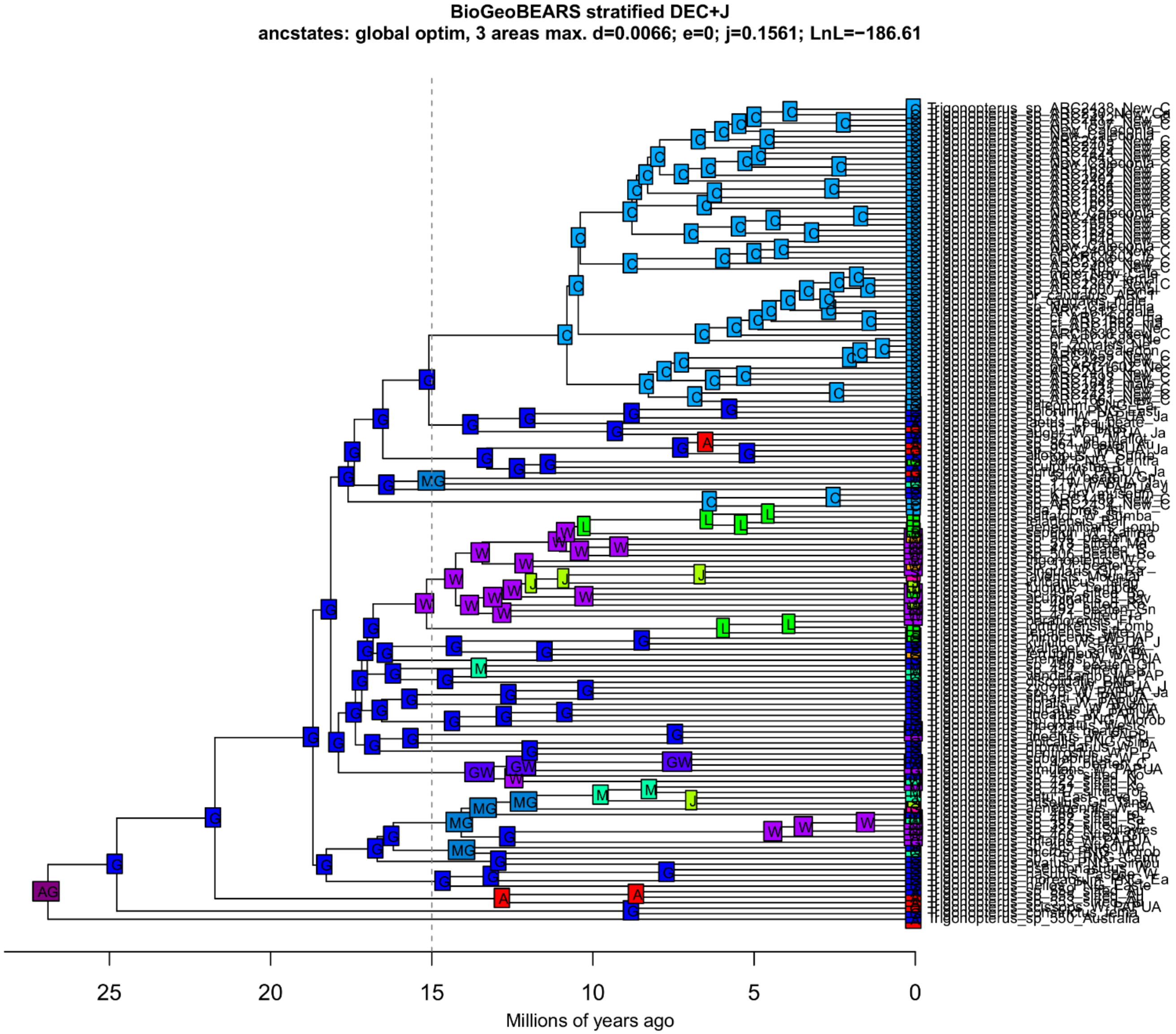
BioGeoBEARS stratified DEC+J
ancstates: global optim, 3 areas max. d=0.0066; e=0; j=0.1561; LnL=−186.61
25
20
15
10
5
0
Millions of years ago
Trigonopterus_sp_550_Australia

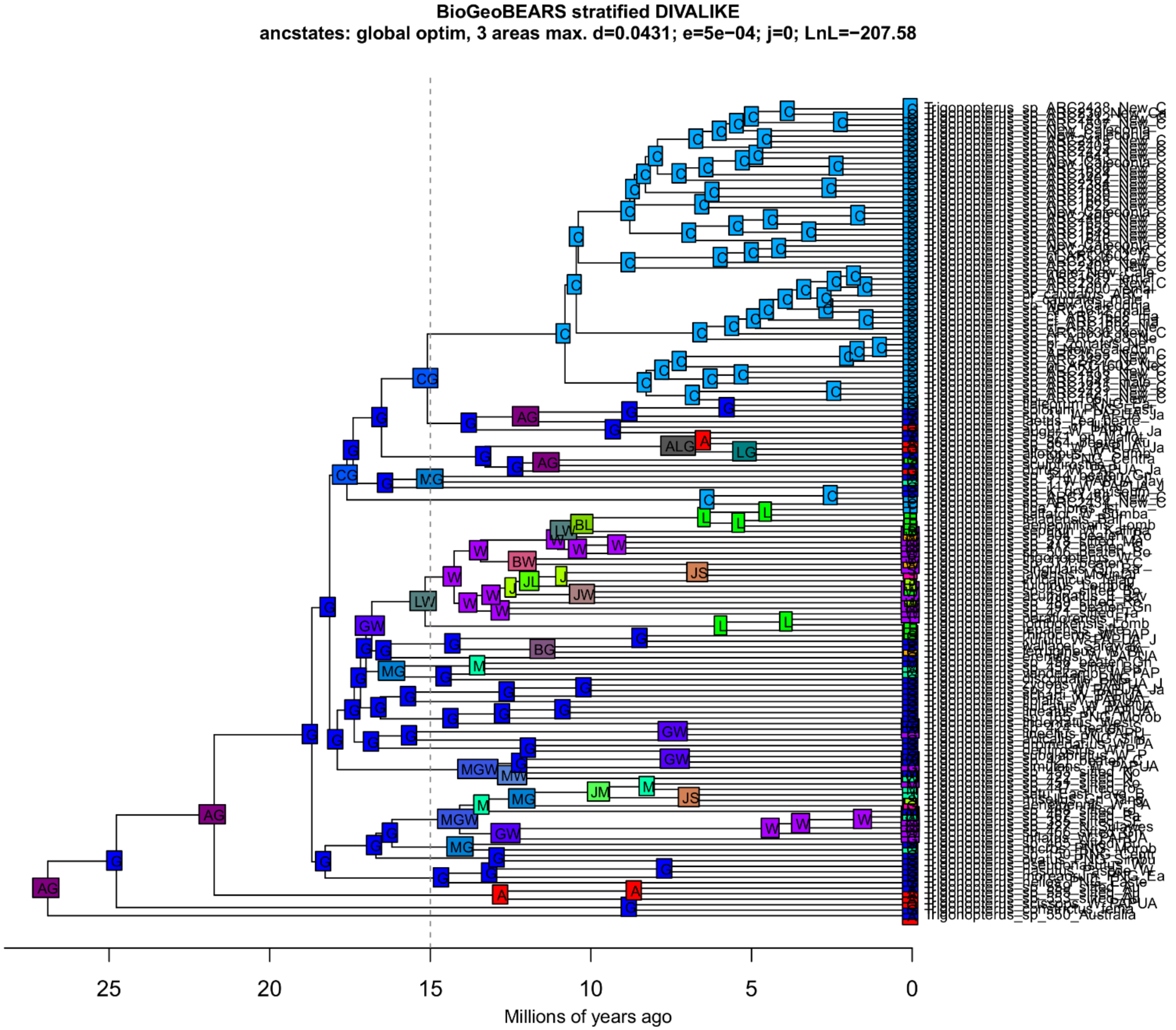

BioGeoBEARS stratified DIVALIKE
ancstates: global optim, 3 areas max. d=0.0431; e=5e−04; j=0; LnL=−207.58
25
20
15
10
5
0
Millions of years ago

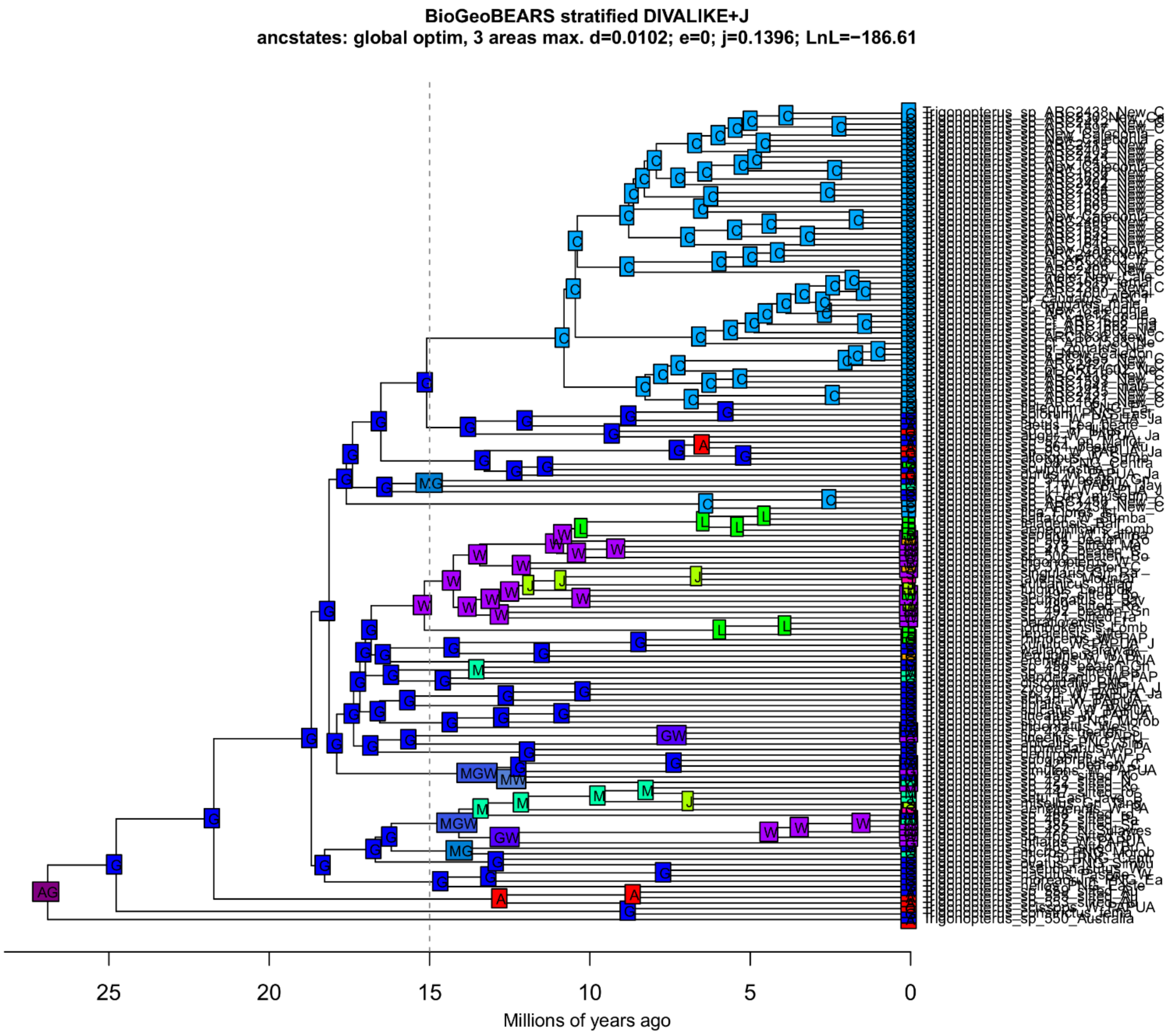

BioGeoBEARS stratified DIVALIKE+J
ancstates: global optim, 3 areas max. d=0.0102; e=0; j=0.1396; LnL=−186.61
Trigonopterus_sp_ARC2438_New_C
Trigonopterus_sp_550_Australia
25
20
15
10
5
0
Millions of years ago

BioGeoBEARS stratified BAYAREALIKE
ancstates: global optim, 3 areas max. d=0.0375; e=0.0351; j=0; LnL=−238.91

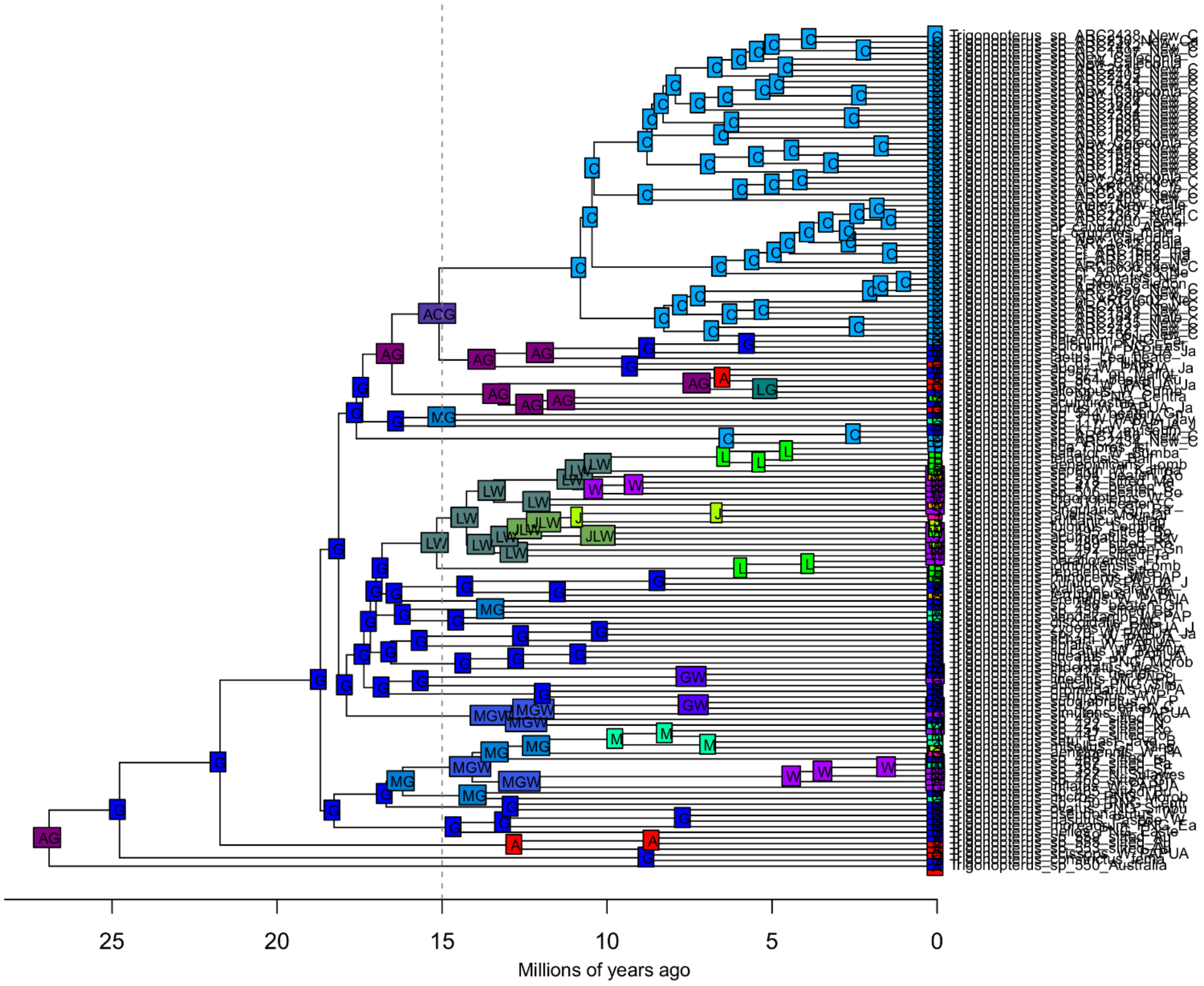

BioGeoBEARS stratified BAYAREALIKE+J
ancstates: global optim, 3 areas max. d=0.001; e=0.001; j=0.1883; LnL=−192.44

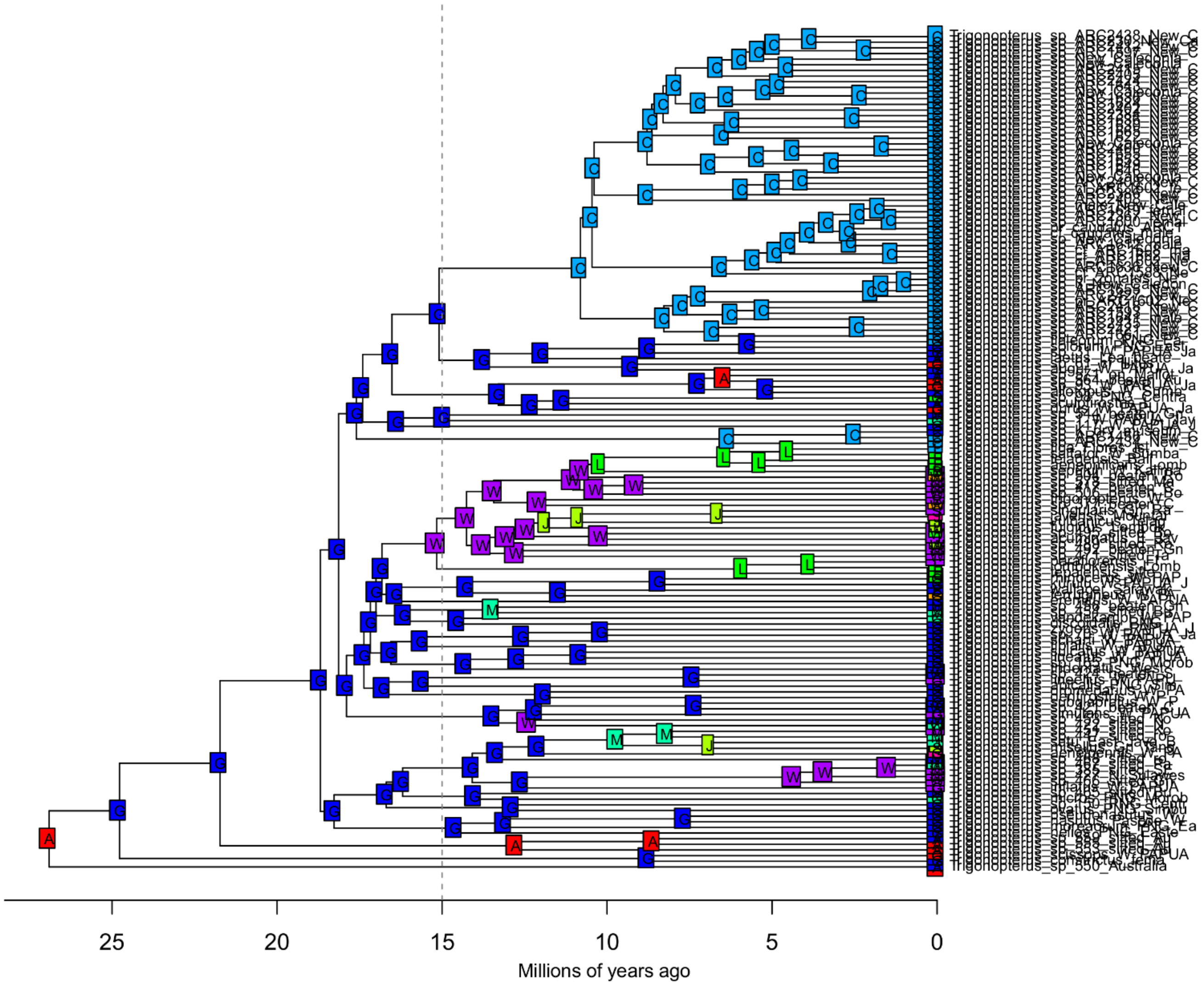

Appendix 6. Detailed result of the DIVALIKE+j analysis conducted in BioGeoBEARS

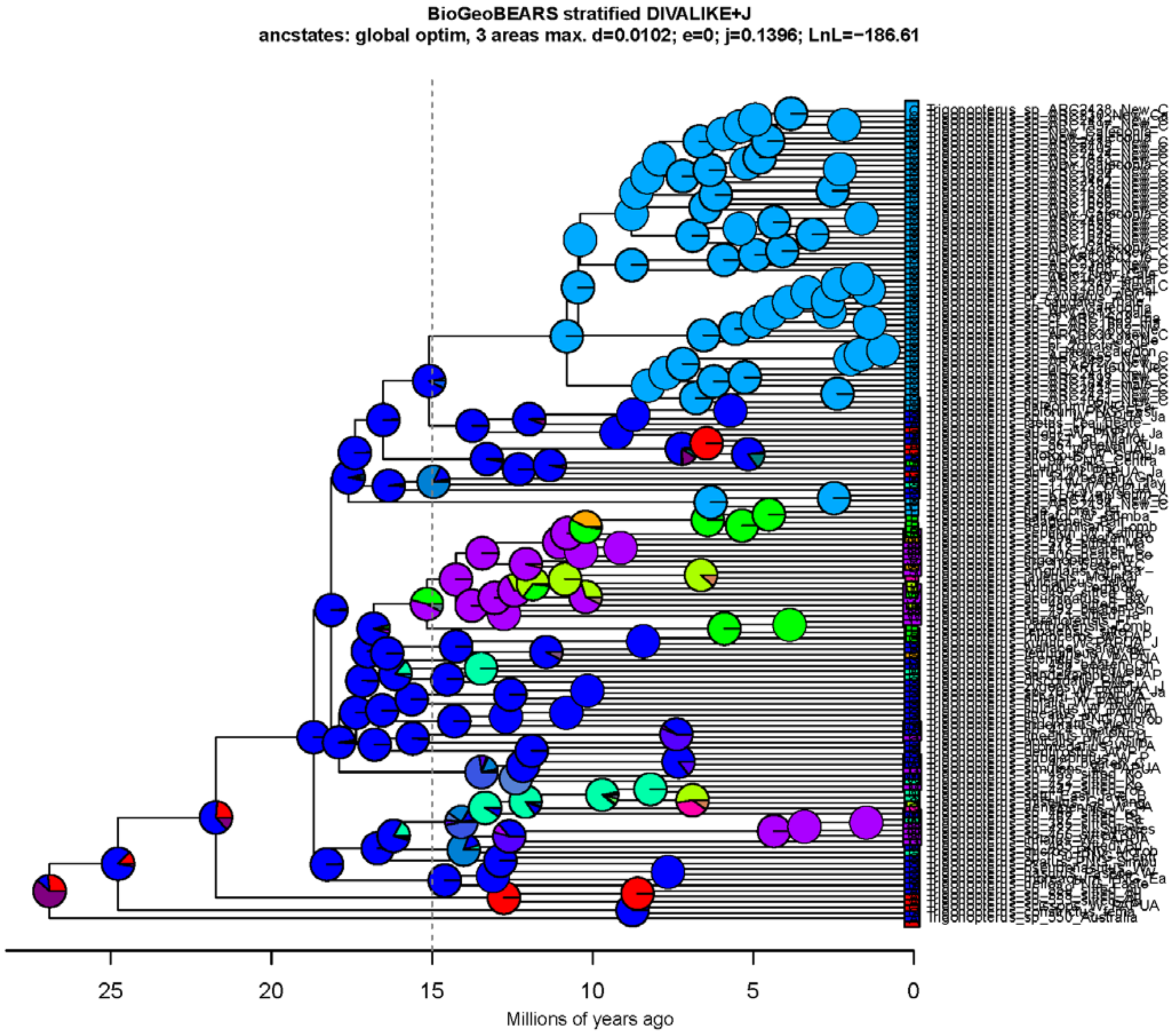

Supplement: Supplementary Information [file rsos160546supp1.pdf]
